# Inhibitory effect of naphthoquine phosphate on *Babesia gibsoni *in vitro and *Babesia rodhaini *in vivo

**DOI:** 10.1186/s13071-021-05127-0

**Published:** 2022-01-07

**Authors:** Shengwei Ji, Mingming Liu, Eloiza May Galon, Mohamed Abdo Rizk, Bumduuren Tuvshintulga, Jixu Li, Iqra Zafar, Yae Hasegawa, Aiko Iguchi, Naoaki Yokoyama, Xuenan Xuan

**Affiliations:** 1 National Research Center for Protozoan Diseases, Obihiro University of Agriculture and Veterinary Medicine, Obihiro, Hokkaido 080-8555 Japan; 2 Department of Microbiology and Immunology, School of Basic Medicine, Hubei University of Arts and Science, Xiangyang, 441053 China; 3 Department of Internal Medicine and Infectious Diseases, Faculty of Veterinary Medicine, Mansoura University, Mansoura, 35516 Egypt; 4 College of Agriculture and Animal Science, Qinghai University, Xining, 810016 China; 5 Joint Department of Veterinary Medicine, Faculty of Agriculture, Tottori University, Tottori, 680-8550 Japan

**Keywords:** Naphthoquine phosphate, *Babesia gibsoni*, *Babesia rodhaini*, In vitro, In vivo

## Abstract

**Background:**

Drug resistance and toxic side effects are major challenges in the treatment of babesiosis. As such, new drugs are needed to combat the emergence of drug resistance in *Babesia* parasites and to develop alternative treatment strategies. A combination of naphthoquine (NQ) and artemisinin is an antimalarial therapy in pharmaceutical markets. The present study repurposed NQ as a drug for the treatment of babesiosis by evaluating the anti-*Babesia* activity of naphthoquine phosphate (NQP) alone.

**Methods:**

An in vitro growth inhibition assay of NQP was tested on *Babesia gibsoni* cultures using a SYBR Green I-based fluorescence assay. In addition, the in vivo growth inhibitory effect of NQP was evaluated using BALB/c mice infected with *Babesia rodhaini*. The parasitemia level and hematocrit values were monitored to determine the therapeutic efficacy of NQP and the clinical improvements in NQP-treated mice.

**Results:**

The half maximal inhibitory concentration of NQP against *B. gibsoni *in vitro was 3.3 ± 0.5 μM. Oral administration of NQP for 5 consecutive days at a dose of 40 mg/kg of body weight resulted in significant inhibition of *B. rodhaini* growth in mice as compared with that of the control group. All NQP-treated mice survived, whereas the mice in the control group died between days 6 and 9 post-infection.

**Conclusion:**

This is the first study to evaluate the anti-*Babesia* activity of NQP in vitro and in vivo. Our findings suggest that NQP is a promising drug for treating *Babesia* infections, and drug repurposing may provide new treatment strategies for babesiosis.

**Graphical Abstract:**

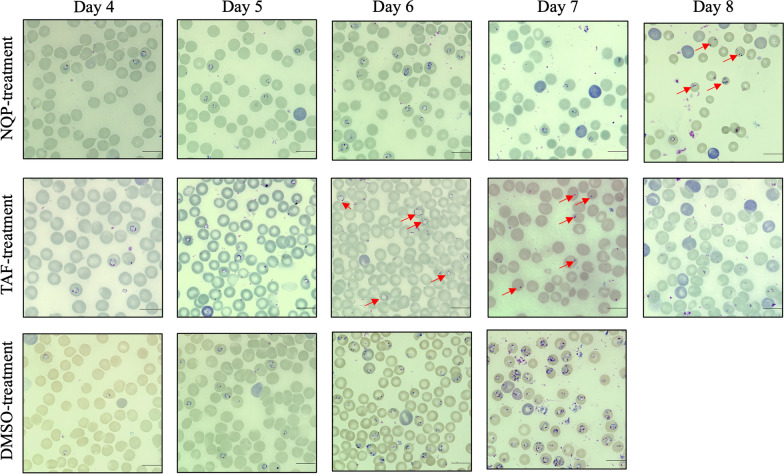

**Supplementary Information:**

The online version contains supplementary material available at 10.1186/s13071-021-05127-0.

## Background

Babesiosis is an infectious disease caused by intraerythrocytic parasites of the genus *Babesia.* More than 100 *Babesia* species have been identified. The most notable species include *Babesia bigemina*, *B. divergens*, and *B. bovis* for bovine, *B. caballi* for equine, *B. canis* and *B. gibsoni* for canine, and *B. microti* and *B. rodhaini* for murine hosts [1]. Moreover, several *Babesia* species have been reported to infect humans. Because of the wide range of hosts, babesiosis is one of the most ubiquitous infections of free-living animals and is attracting increasing interest as an emerging zoonosis in humans [1]. Parasites replicate in the mammalian host red blood cells (RBCs) and produce clinical symptoms including fever, hemolytic anemia, anorexia, hemoglobinuria, and emaciation, and the severe infection phase often results in death [2]. Current treatment strategies for babesiosis are limited. For instance, imidocarb dipropionate and diminazene aceturate are used for the treatment of animal babesiosis, and atovaquone combined with azithromycin and clindamycin combined with quinine are recommended for the treatment of human babesiosis [3]. However, adverse side effects of imidocarb dipropionate, diminazene aceturate, and quinine, low effectiveness of clindamycin or azithromycin, and the emergence of drug resistance to atovaquone are well documented [4–9].

Over the past five decades, cases of human babesiosis have increased in the United States [10]. A review study reported that during the period from 1982 to 1993, 139 hospitalizations occurred due to *Babesia* infection. Among the patients, 25% required intensive care stays and nine patients died [11]. Moreover, recent surveillance in the USA found that a total of 7612 cases of babesiosis were reported to the Centers for Disease Control and Prevention (CDC) from 2011 to 2015. Of 7612 cases, 82.5% were classified by the reporting health jurisdiction as confirmed babesiosis and 17.5% as probable. Among these patients, 7 of 46 deaths were attributed to babesiosis and 4 of 46 deaths were not babesiosis-related, whereas for 35 patients, whether the deaths were due to babesiosis was not verified [12]. Atovaquone combined with azithromycin as the standard therapy for treating human babesiosis has failed in some clinical cases, caused by a single nucleotide polymorphism (SNP) in the *B. microti* cytochrome b gene [5]. In addition, although the atovaquone–azithromycin drug combination is effective on *B. gibsoni* infections, a recent study found that 8.57% of *B. gibsoni* isolates obtained from Japanese dogs carry a SNP in the cytochrome b gene, which was empirically proven to be associated with resistance to atovaquone [13, 14]. Therefore, new drugs are needed to combat the emergence of drug resistance and to develop effective treatment options.

Naphthoquine (NQ) is an antimalarial drug that was first synthesized in China in 1986 and registered as naphthoquine phosphate (NQP) in 1993. Initial clinical trials showed that NQ monotherapy was highly efficacious without documented toxicity [15, 16]. Subsequently, a drug combination comprising NQ and artemisinin (ART) at a fixed ratio of 1:2.5 was developed in order to retain the strongpoint of the two drugs and for prevention of the possible emergence of drug resistance. Safety data for NQ-containing therapies involving more than 4000 patients showed no serious adverse reaction, hematology, or biochemistry changes. It has been considered as a promising antimalarial drug candidate and is marketed under the name of ARCO^®^ in various tropical countries [15–17]. Because of the close relationship between *Plasmodium* and *Babesia* genera, we investigated whether NQP has effects on the in vitro growth of *B. gibsoni*, a causative agent of canine babesiosis, and in vivo-propagated *B. rodhaini*, a highly pathogenic rodent *Babesia* species.

## Methods

### Chemicals

NQP was purchased from ChemScene (NJ, USA), and was dissolved in dimethyl sulfoxide (DMSO, Sigma-Aldrich, Tokyo, Japan) to prepare a 40 mg/ml stock solution. In parallel, tafenoquine (TAF, Sigma-Aldrich, Tokyo, Japan) was dissolved as mentioned above, and was used as a control treatment. TAF was previously reported as a potent anti-*Babesia* agent for treating *B. rodhaini* infection [18]. SYBR Green I (SG1) nucleic acid stain was purchased from Lonza America (GA, USA).

### Maintenance of the parasites in vitro and in vivo

The *Babesia gibsoni* Oita strain [19] was cultured and used for the in vitro growth inhibition assay. For maintenance, the *B. gibsoni* was cultured in canine RBCs and was suspended in a culture medium, RPMI-1640, supplemented with 20% canine serum. The culture was maintained in an atmosphere of 5% CO_2_ and 5% O_2_.

For the in vivo inhibition assay, the *B. rodhaini* Australia strain [20] was recovered from the stock in our laboratory. For the maintenance of *B. rodhaini*, cryopreserved parasitized RBCs were passaged by intraperitoneal (i.p.) injection of mice. Challenge infection was performed with i.p. inoculation of 10^7^ fresh *B. rodhaini*-infected RBCs (iRBCs). A total of 20 BALB/c mice (6 weeks old) were purchased from CLEA Japan and were used to maintain *B. rodhaini* for the in vivo study.

### In vitro growth inhibition assay

To test the growth inhibitory effect of NQP on *B. gibsoni*, a SG1 fluorescence-based assay was performed as reported previously [21]. Briefly, the in vitro cultures of *B. gibsoni* were diluted to 1% parasitemia with fresh canine RBCs. The stock solution of NQP was diluted in medium to achieve final concentrations of 0.1, 0.5, 1.0, 2.5, and 5.0 μM, and incubated with iRBCs in triplicate in 96-well plates with 5% hematocrit (HCT) for 96 h. After a lysis buffer containing a 2 × SG1 nucleic acid stain was added in each well, the fluorescence values were evaluated using a fluorescence spectrophotometer (485 and 518 nm, Fluoroskan Ascent, Thermo Fisher Scientific, USA) and the inhibitory activity and half maximal inhibitory concentration (IC_50_) values were calculated using GraphPad Prism 8 (GraphPad Software Inc., USA).

### In vivo growth inhibition assay

Fifteen BALB/c mice intraperitoneally challenged with 10^7^ *B. rodhaini* (iRBCs) were randomly assigned to groups (*n* = 5 per group). When parasitemia was about 3–5%, the drug treatment was initiated. The first group was treated orally with 40 mg/kg of NQP for 5 consecutive days as previously described [22, 23]. The second group was treated orally with 20 mg/kg TAF as single-dose therapy, according to a previously regimen [18]. The control group was treated orally with 5% DMSO in Milli-Q water. Parasitemia was calculated from Giemsa-stained blood smears by counting infected RBCs among 3000 RBCs. HCT changes were monitored for the development of an index of anemia by using a hematology analyzer (Nihon MEK-6450, Nihon Kohden Corporation, Tokyo, Japan) every 2 days until 42 days post-infection (dpi). All experiments were approved by the Animal Welfare Committee (approval no. 20-128) and were conducted in accordance with the standards for the care and management of experimental animals as stipulated by the Obihiro University of Agriculture and Veterinary Medicine, Hokkaido, Japan.

### Statistical analysis

Data analysis was performed using GraphPad Prism. Differences in parasitemia between the control and treated groups were determined by one-way analysis of variance (ANOVA) plus Tukey–Kramer post hoc analysis. Survival rates were calculated using the Kaplan–Meier method, with regard to the log-rank test. A *P*-value < 0.05 was considered statistically significant.

## Results

### Effects of NQP on *B. gibsoni* growth in vitro

**Fig. 1:**
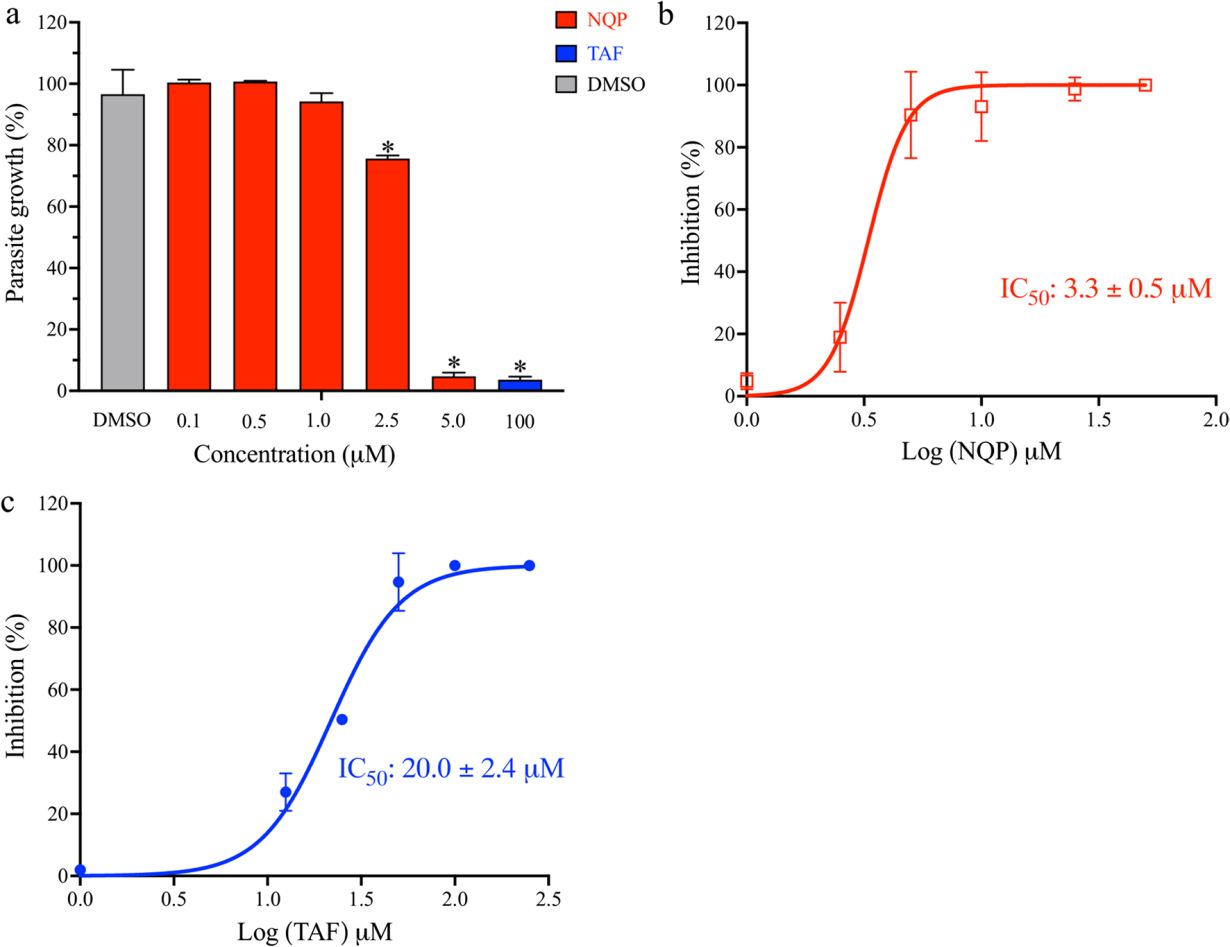
The in vitro growth inhibitory effect of naphthoquine phosphate (NQP). **a** NQP inhibits growth of *B. gibsoni *in vitro. The gray column represents the DMSO-treated culture as a drug solvent control, the red columns represent the NQP-treated cultures, and the blue column represents 100 μM tafenoquine (TAF)-treated culture. **b** Dose-dependent inhibition curve of NQP on *B. gibsoni *in vitro. **c** Dose-dependent inhibition curve of TAF on *B. gibsoni *in vitro. Each value represents the mean ± standard deviation (SD) of three independent experiments carried out in a triplicate. The asterisks indicate a significant difference (*P* < 0.05) between the drug-treated cultures and the DMSO-treated culture

NQP significantly inhibited *B. gibsoni* growth at 2.5 μM and 5 μM (Fig. 1a) (*F*_(6,14)_ = 552.9, *P* < 0.05). The IC_50_ value of NQP on *B. gibsoni* was 3.3 ± 0.5 μM (Fig. 1b). Meanwhile, the IC_50_ value of TAF was 20.0 ± 2.4 μM (Fig. 1c).

### Effects of NQP on *B. rodhaini* in vivo

**Fig. 2:**
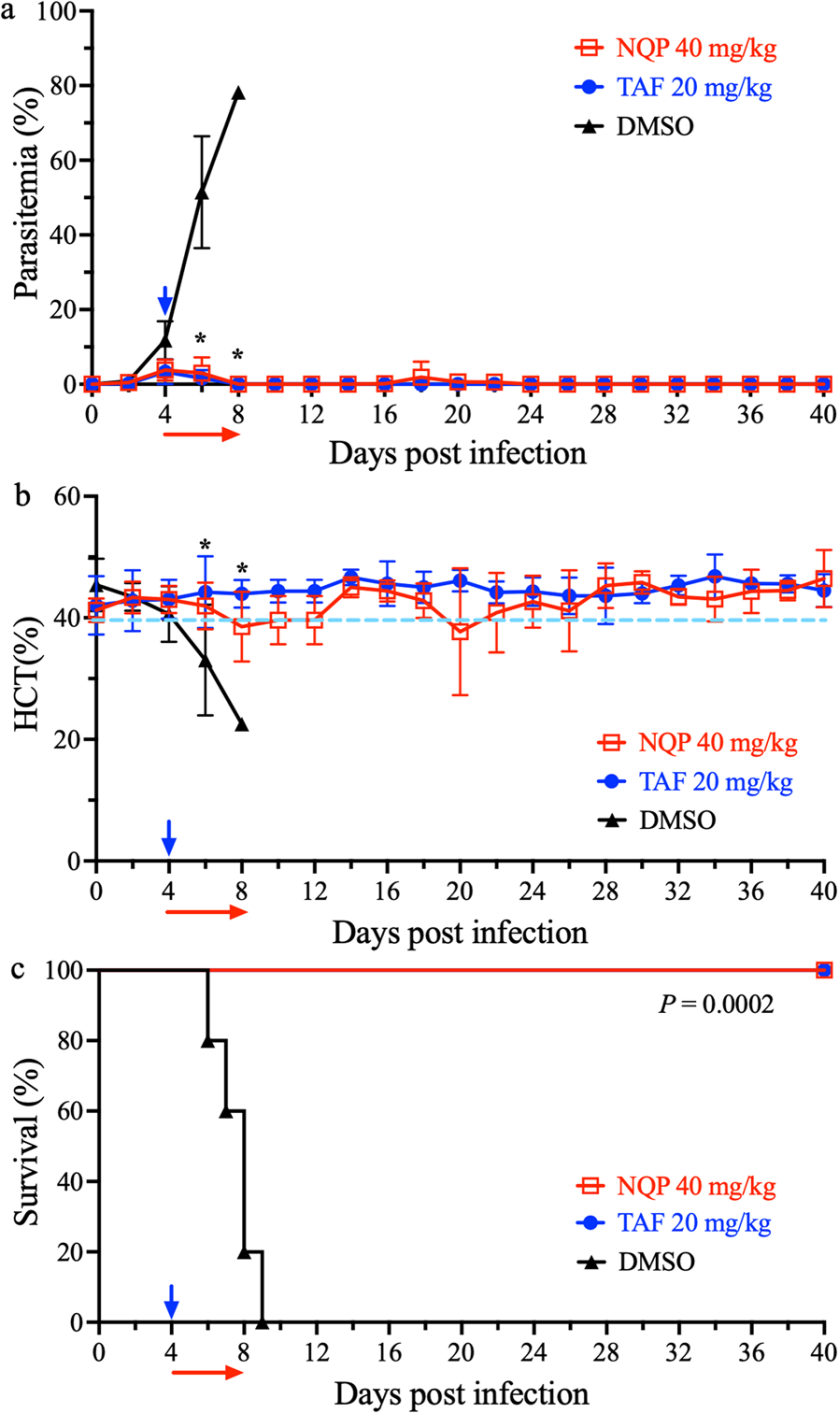
The growth inhibitory effect of naphthoquine phosphate (NQP) on *B. rodhaini* in BALB/c mice. **a** NQP and tafenoquine (TAF) prevent the typical growth of *B. rodhaini* in mice as compared with that in DMSO-treated mice as a drug solvent control. **b** Changes of hematocrit (HCT) values in mice treated with NQP or TAF as compared with that in DMSO-treated mice. The asterisks indicate a significant difference (*P* < 0.05) between the NQP- or TAF-treated group and the DMSO-treated group. **c** Survival rates of NQP-, TAF-, and DMSO-treated mice. The arrows indicate time of treatment. Parasitemia was calculated by counting infected RBCs among 3000 RBCs using Giemsa-stained blood smears. Dotted line indicates the reference range

The highest parasitemia of *B. rodhaini* in DMSO-treated group was 78.2% at 8 dpi (Fig. 2a). In contrast, the parasitemia in NQP- and TAF-treated groups were decreased after treatment and showed 95.1% (3.8% peak parasitemia) and 95.8% (3.3% peak parasitemia) inhibition compared to the highest parasitemia in the DMSO-treated group, respectively. A significant difference in parasitemia levels was calculated in NQP-treated group and TAF-treated group as compared with the DMSO-treated group at 6 and 8 dpi (*F*_(2, 44)_ = 15.87, *P* < 0.05). Parasitemia was undetectable via Giemsa-staining in mice treated with NQP and TAF at 10 dpi and 8 dpi, respectively. Afterwards, regrowth of parasites was observed in both NQP-treated (*n* = 2/5) and TAF-treated (*n* = 1/5) groups at 16 dpi and 22 dpi (Fig. 2a), respectively. Significant reductions in HCT values were observed in the DMSO-treated mice at 6 dpi and 8 dpi as compared to the values recorded from the NQP- or TAF-treated group (Fig. 2b) (*F*_(2, 24)_ = 10.35, *P* < 0.05). All the mice in the NQP- and TAF-treated groups survived until the end of the experiment (by 40 dpi; *P* = 0.0002), whereas none of DMSO-treated mice survived by 10 dpi (Fig. 2c). Compared with the DMSO-treated group, the TAF-treated parasites showed abnormal morphological changes such as faint chromatin staining and degenerative forms from 5 dpi onwards, while denigrative forms of NQP-treated parasites were observed at 8 dpi (Additional file 1: Figure S1).

## Discussion

NQ is a 4-aminoquinoline antimalarial drug with a longer half-life but slower action than ART, and is currently combined with ART to treat malaria [15]. This combination therapy is also effective on *Schistosoma mansoni* [24]. NQP combined with azithromycin for malaria treatment is available in the market [25]. The toxic effects of NQP on mammalian hosts have been reported. Daily treatments in dogs for 14 days at a dose of 17.5 mg/kg/day and in rats for 70 mg/kg/day were safe. The safe doses in canine and rat models are equivalent to approximately 10 mg/kg/day in humans [16, 26]. A previous study reported that the concentration of NQP reached the peak level in plasma 2 h post treatment with a dose of 10 mg/kg. The peak concentrations were 300.84 ng/ml and 273.29 ng/ml in plasma and erythrocytes, respectively, and the half-life of NQP was 198 h (~ 8 days) in normal mice [27]. Interestingly, the concentrations were far greater in *Plasmodium berghei*-infected mice [27]. The antiparasitic activity of NQP, as shown by the inhibition of *B. gibsoni *in vitro (Fig. 1) prompted us to further explore its anti-*Babesia* activity in vivo. We used the lethal species *B. rodhaini* in the mouse model for evaluating NQP as a therapeutic*.* In the current in vivo trial, NQP exhibited excellent inhibitory efficacy as evidenced by reduced parasite growth (Fig. 2a) and degenerative morphological changes in the parasites (Additional file 1: Figure S1). Furthermore, the first 2 days of treatment with 40 mg/kg NQP prevented the rise of *B*. *rodhaini* parasitemia starting from 6 dpi compared with the typical rise of mean parasitemia in DMSO-treated mice. In addition, the accumulation of NQP in plasma [27] by completion of the 4-day treatment resulted in morphological changes of parasites in all treated mice at 8 dpi. The TAF-treated group showed an aberrant parasite phenotype (Additional file 1: Figure S1) which has been associated with oxidative stress [18]. *Babesia rodhaini*-infected mice in the DMSO-treated group rapidly developed anemia, whereas NQP and TAF prevented anemia development in infected mice (Fig. 2b).

Since TAF was approved by the US Food and Drug Administration (FDA) as a single-drug treatment for malaria, TAF studies have attracted much attention [18, 22]. The limitation of TAF is the risk of inducing severe hemolytic anemia in individuals with G6PD deficiency in humans and relapse of parasitemia, which are well documented [18, 28, 29]. A single treatment of TAF on immunocompromised hosts could not eliminate parasites [18, 28]. Recently, TAF showed strong and broad antiparasitic activity against *Babesia* spp., including *B. microti*, *B. gibsoni*, and *B. rodhaini* [18]*.* Hence, TAF was selected as a reference drug in this study. In the present study, the relapse of parasitemia was observed in both the NQP-treated group and TAF-treated group (Fig. 2a). Therefore, NQP may need to be accompanied by other anti-*Babesia* drugs to augment its effect and prevent the regrowth of parasites.

In addition, the mechanism of action of NQP has not been fully elucidated. The inhibitory activity of NQP for *Plasmodium* was hypothesized to be through the inhibition of hemozoin bio-crystallization in the digestive vacuole of late-stage parasites and disruption of the membrane system. Due to *Babesia* not producing hemozoin during parasite development, the inhibitory effect of NQP on the *Babesia* parasite is hypothesized to be related to targeting the parasite’s membrane system [16, 30].

It should be noted that there are some limitations to the present study. Although NQP exhibited a potential anti-*Babesia* effect, it has a slower onset of action and a longer half-life, which may easily lead to drug build-up with increasing the probability of developing resistance. Therefore, future studies are warranted to analyze the possible synergistic effect of NQP when administrated in combination with other drugs which have a rapid onset of babesicidal action and a short half-life. Such analysis will help to determine the most effective composition ratio for treatment of *Babesia* in animals in clinical applications. Furthermore, the mode of action by which NQP inhibits the in vitro and in vivo growth of *Babesia* is still unknown. Consequently, further studies are required to elucidate this point. Although the present study demonstrated the potential anti-*Babesia* efficacy of NQP in a mouse model, additional in vivo experiments are required to confirm such inhibitory effect in *B. gibsoni*-infected dogs.

## Conclusions

The present study demonstrated the growth inhibitory effect of NQP against *B. gibsoni *in vitro and *B. rodhaini *in vivo*.* Our findings indicate that NQP is a potential candidate agent for the treatment of babesiosis and suggest further investigation on the possible use of this chemical for canine babesiosis and human babesiosis.

## Supplementary Information

**Additional file 1: Figure S1.** Light micrographs of *B. rodhaini-*infected mice during NQP and TAF treatment (from 4 to 8 dpi) and of DMSO-treated mice (from 4 to 7 dpi). Compared with the DMSO-treated group, NQP treatment exhibits degenerated parasites at 8 dpi (red arrow), whereas parasites in the TAF-treated mice show a vacuole-like aberrant phenotype. Bars = 10 μm.

## Data Availability

All data sets were presented as tables, figures and text description in this article.

## Abbreviations

NQ: Naphthoquine
NQP: Naphthoquine phosphate
ART: Artemisinin
TAF: Tafenoquine
SG1: SYBR Green I
DMSO: Dimethyl sulfoxide
RBCs: Red blood cells
iRBCs: Infected red blood cells
i.p.: Intraperitoneal
IC_50_: Half maximal inhibitory concentration
SD: Standard deviation
dpi: Days post-infection
HCT: Hematocrit
