# Supplementary figures and images for: Inhibitory effect of naphthoquine phosphate on Babesia gibsoni in vitro and Babesia rodhaini in vivo
Source: Parasit Vectors. 2022 Jan 7;15:10. doi: 10.1186/s13071-021-05127-0 (PMC8740460; doi:10.1186/s13071-021-05127-0)

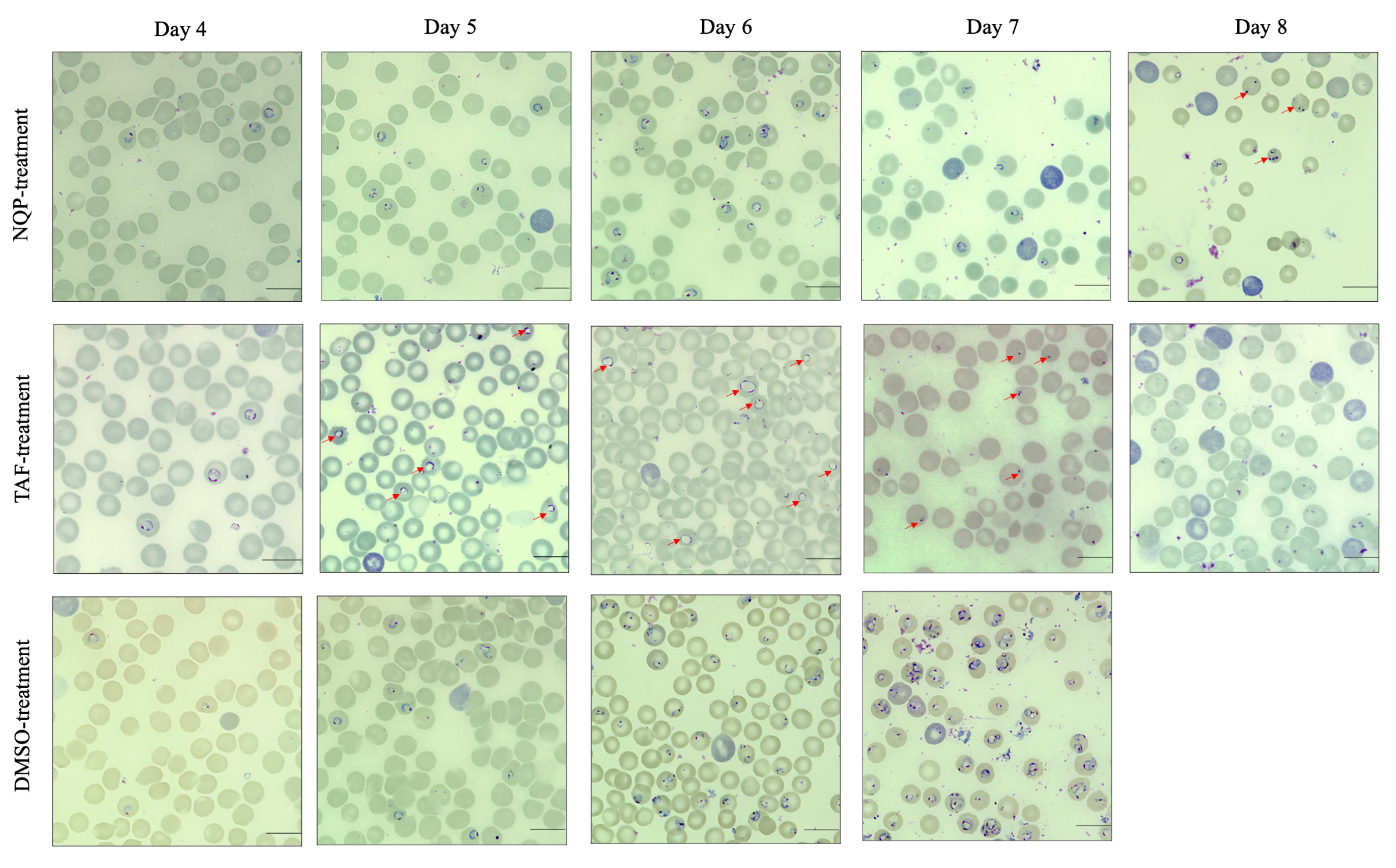

Supplement: Supplementary file 1 — Additional file 1: Figure S1. Light micrographs of B. rodhaini-infected mice during NQP and TAF treatment (from 4 to 8 dpi) and of DMSO-treated mice (from 4 to 7 dpi). Compared with the DMSO-treated group, NQP treatment exhibits degenerated parasites at 8 dpi (red arrow), whereas parasites in the TAF-treated mice show a vacuole-like aberrant phenotype. Bars = 10 μm. [file 13071_2021_5127_MOESM1_ESM.tif]
